# Real-space modeling for complex structures based on small-angle X-ray scattering

**DOI:** 10.1107/S1600576721006701

**Published:** 2021-08-18

**Authors:** Kazuhiko Omote, Tomoyuki Iwata

**Affiliations:** a X-ray Research Laboratory, Rigaku Corporation, 3-9-12 Mastubara-cho, Akishima, Tokyo 196-8666, Japan

**Keywords:** small-angle X-ray scattering, SAXS, hierarchical structures, reverse Monte Carlo, aerogels, computer simulations

## Abstract

A three-dimensional real-space model has been created for hierarchical materials by matching observed and simulated small-angle X-ray scattering patterns. The simulation is performed by arranging the positions of small primary particles and constructing an aggregate structure in a finite-sized cell. In order to avoid the effect of the finite size of the cell, the cell size is extended to infinity by introducing an asymptotic form of the long-range correlations among the primary particles. As a result, simulations for small-angle X-ray scattering patterns can be performed correctly in the low-wavenumber regime (<0.1 nm^−1^), allowing the model to handle hundred-nanometre-scale structures composed of primary particles of a few nanometres in size. An aerogel structure was determined using this model, resulting in an excellent match with the experimental scattering pattern. The resultant three-dimensional model can generate cross-sectional images similar to those obtained by transmission electron microscopy, and the calculated pore-size distribution is in accord with that derived from the gas adsorption method.

## Abstract 2

A three-dimensional real-space model has been created for complex hierarchical materials by matching observed and simulated small-angle X-ray scattering patterns. The simulation is performed by arranging the positions of small primary particles and constructing an aggregate structure in a finite-sized cell.

## 1. Introduction   

Many functional materials have a hierarchical structure, *e.g.* catalyst carriers, hybrid polymer composites, high-performance rubber tires, aerogels and so forth. They are composed of complex formations of atomic-scale or nanometre-sized primary units. Key functional performance is closely related to the characteristics not only of the primary unit but also of the configuration and formation of the units. Transmission and scanning electron microscopy (TEM and SEM, respectively) are powerful tools to investigate the precise structure of these primary units; however, it is difficult to investigate such hierarchical complex structures using TEM and SEM because the materials can be easily destroyed during sample preparation, *e.g.* slicing and thinning. On the other hand, the X-ray scattering method can be used for nondestructive analysis of structures that range in size from sub-nanometre to a few hundred nanometres due to the lower absorption of X-rays, which can penetrate through the functional materials as is.

Small-angle X-ray scattering (SAXS) is commonly used to analyze the size and size distribution of structures in the nanometre region (Guinier & Fournet, 1955 ▸). To investigate complex structures such as aerogels, Hasmy *et al.* (1994 ▸) and Hasmy & Jullien (1994 ▸) developed a structure factor model based on real-space numerical simulation. However, it was assumed that the constituent primary particles were identical, and the structure factor could be factored from the form factor. This simulation exhibits oscillatory fringes corresponding to the size (diameter) of the primary particles. As usual, the experimental scattering patterns do not show such fringes because the real physical system is polydisperse. Therefore, a precise comparison between the experimental and simulated scattering intensities could not be performed without introducing polydispersity of the primary particles (distribution of particle size).

Recently, computer calculation power has increased, making it possible to simulate X-ray scattering intensity with a model that includes more than 100 000 primary units. We have built a model structure with various cell sizes, using spherical particles as the primary unit. We assume a spherical shape for the particles for the sake of simplicity, but introduce a size distribution in order to fit the experimental scattering intensity with that of the simulation in the entire regime of the measured SAXS pattern. Optimization of the model is performed using the Monte Carlo method (McGreevy & Pusztai, 1988 ▸; Keen & McGreevy, 1990 ▸). We moved the position of a particle randomly and simulated the scattering intensity. The result is compared with experimental data to decide whether the movement should be selected or not, taking into account the improvement in the error. This procedure is repeated numerous times for all the particles until the error does not decrease any more.

The cell size effect for the simulated intensity should be noticeable in the low-*q* regime [wavenumber *q* = (4π/λ)sinθ, where θ is half the scattering angle and λ is the wavelength of the incident radiation]. The simulated intensity oscillates according to the shape of the cell, heavily affecting the intensity of the small-angle scattering. Introducing a periodic boundary condition does not help to reduce this artificial oscillation. In order to overcome the problem, we extended the cell size to infinity by introducing an asymptotic form for long-distance particle–particle correlations. As a result, we can simulate the small-angle scattering regime below 0.05 nm^−1^ without any size effect for real-space boundaries with a finite number of particles.

In the next section, details of the presented simulation method are described. In the subsequent section, we will present a structural analysis of aerogel materials, which are of considerable interest as functional materials due to features such as ultra-low density, low thermal conductivity and high flexibility (Gurav *et al.*, 2010 ▸). Aerogels contain many nanometre-sized pores and have a complex structure (Kanamori, 2011 ▸) that is difficult to investigate precisely. Our proposed technique can determine a real-space structure fitted to the experimental SAXS pattern. The obtained structural model enables the study of numerical simulations and the correlation of the nanometre-scale structure with various physical properties. Finally, we present a discussion and summary.

## 2. Simulation of X-ray scattering intensity based on the real-space structure   

**Figure 1:**
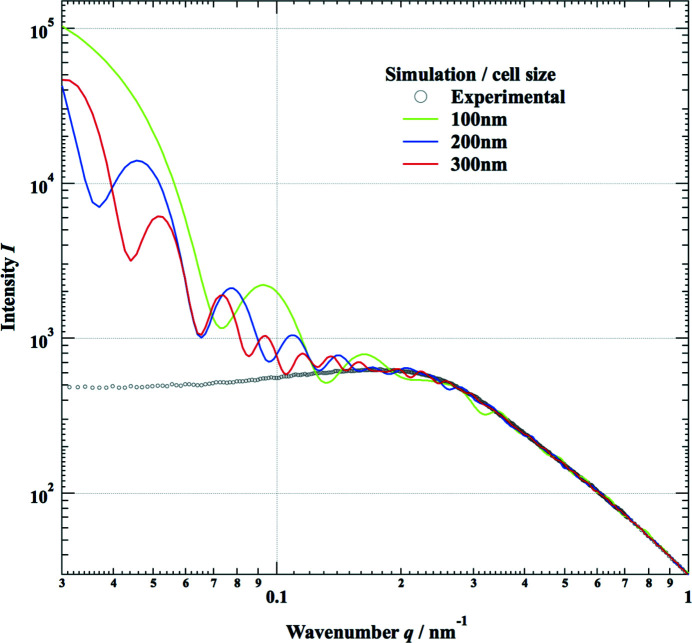
Simulated small-angle scattering patterns based on the Debye scattering equation with cell sizes of 100, 200 and 300 nm. In the low-*q* regime, the calculated intensities are much higher than the experimental results due to the shape factor of the calculated cells.

The X-ray scattering intensity in electron units of a scattering vector **q** = **k**_out_ − **k**_in_ (**k**_in_ and **k**_out_ are the wavevectors of the incident and scattered X-rays, respectively) for a particle system composed of *N* independent particles is calculated via the following function (Warren, 1990 ▸):
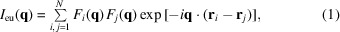
where *F*_*i*_(**q**) [*F*_*j*_(**q**)] is the form factor of the *i*th (*j*th) particle, and **r**_*i*_ (**r**_*j*_) is the positional coordinate of the *i*th (*j*th) particle. If we assume an isotropic system and average the orientation of **q**, then equation (1) can be written as a function of *q* = |**q**|,
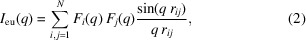
where *r*_*ij*_ = |**r**_*i*_ − **r**_*j*_|. This is called the Debye scattering equation (Debye, 1915 ▸). As mentioned in the *Introduction* [Sec sec1], when we calculate a scattering pattern based on equation (2) for a finite cell size (limited *r*_*ij*_ range), we observe oscillating fringes in the low-*q* region (*q* < 0.5 nm^−1^), as shown in Fig. 1 ▸. The curves in Fig. 1 ▸ are calculated with a cell size *L* of 100, 200 or 300 nm, and the period of the fringes corresponds to the cell size of the simulation. This is a serious problem for simulating and investigating the complex structures of composite materials that are several tens of nanometres in size because the small-angle scattering pattern in this region has an essential role in determining those structures. Of course, the real system is much larger than a few hundred nanometres and no fringes are observed in the real experimental scattering patterns. In order to avoid such unphysical behavior in the simulation, the most direct solution is to increase the cell size of the simulation. However, if we increase the number of particles *N*, the calculation time will increase proportionally by *N* ^2^ (number of correlations) and the volume of the cell increases with *L* ^3^. This means that the calculation time of the structural simulation is proportional to *L* ^6^, and it is not realistic to increase the cell size sufficiently to attenuate the unphysical fringes, particularly in the extremely low *q* region (*q* < 0.1 nm^−1^). Therefore, we have taken another approach described as follows.

Equation (2) can be calculated separately in two parts by introducing a long distance *R* as follows:
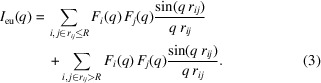
When *R* satisfies the condition that there are many particle pairs in the same distance range *r*_*ij*_, we assume that the individual form factor pairs can be replaced with the average value in the second term of equation (3):
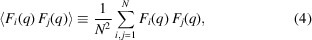


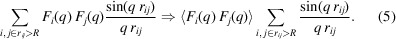
When we introduce the number density ρ_*i*_(*r*_*ij*_) of the particles at a distance *r*_*ij*_ relative to the *i*th particle, the summation over the *j*th particle in equation (5) can be replaced by an integral:
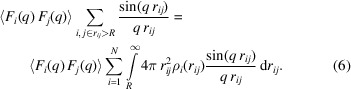
After averaging ρ_*i*_(*r*_*ij*_) for all particles *i*, 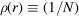
 × 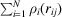, equation (6) is written as
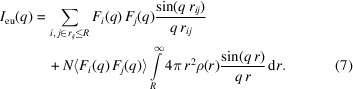



If the integration range of *r* in equation (7) is finite, the simulated SAXS pattern will have oscillations in the small-angle regime as shown in Fig. 1 ▸. Therefore, we have extended the integration range of *r* to infinity by assuming an appropriate value ρ(*r*). The purpose of the simulation for the SAXS pattern is to investigate density fluctuations in the system. This means that the simulation cell size *L* must be large enough to determine the specific structure. Beyond this specific scale, it should be reasonable to assume
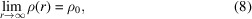
where ρ_0_ is the average particle density over the entire system. From a numerical simulation point of view, the cell size *L* must be large enough to satisfy ρ(*r* ≃ *L*/2) ≃ ρ_0_ in order to configure the correct particle arrangement in the cell that is consistent with the SAXS pattern (*L*/2 is the maximum inter-particle distance to avoid double counting of the same particle pair in the simulation). Then, outside of the cell in equation (7), we can continue to integrate with ρ(*r*) = ρ_0_ and it becomes
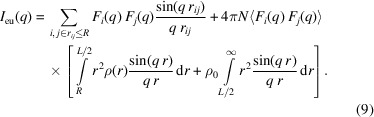



To fit the experimental SAXS pattern, we also have to take into account the resolution of the measurement instrument. If we assume the resolution is isotropic and has the same value in any direction, then equation (1) can be modified by the convolution of divergent **q** vectors as follows:
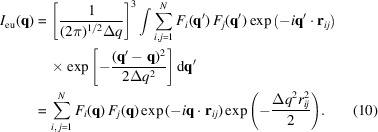
For this derivation, we assume that the form factor of the primary particle varies slowly and can be set to *F*_*i*_(**q**) ≃ *F*_*i*_(**q**′) during the integration. For example, for a practical SAXS instrument using a two-dimensional detector, the resolution is limited by the spot size on the detector δ_D_ and camera length *C*_L_. The angular resolution of the system is δ_D_/*C*_L_ and is estimated to be
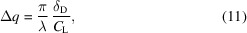
where λ is the X-ray wavelength. Equation (10) can be applied to equation (9) and we finally obtain
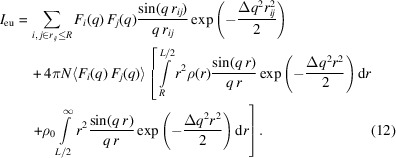



The third term of equation (12) is considered to be constant during the simulation after we determine the parameters ρ_0_, *L* and Δ*q*, and the integration converges much faster without considering the coherence length (resolution) of the experimental system. Therefore, we can simulate the scattering patterns of a complex configured system based on equation (12) free from the problems of a finite cell size.

## 3. Structural determination based on reverse Monte Carlo simulation for an aerogel material   

Aerogels, known to be some of the lowest-density solid materials, have attracted attention as insulators due to their very low thermal conductivity. They are composed of many nanometre-sized pores, and the air in the pores cannot move smoothly so convection is suppressed. In addition, because the solid framework represents a small portion of the material, they are ineffective for conducting heat. These physical properties are closely correlated to features of the primary units and their network structures. However, TEM (or SEM) is not suitable to investigate the entire three-dimensional structure of these materials because it can measure only very thin samples (or the surface of the samples) and the depth information may be destroyed during sample preparation.

Therefore, nondestructive SAXS (and neutron scattering) studies have been performed by many authors (Kanamori *et al.*, 2009 ▸; Wu & Lin, 2012 ▸). In order to investigate structures having a complex formation of primary particles by SAXS, fractal dimension analysis has often been discussed (Beaucage, 1996 ▸) and the structures have been characterized by the slopes of the SAXS patterns in specific *q* regimes (Grigoriew & Gronkowski, 2006 ▸; Grigoriew *et al.*, 2008 ▸). These approaches are simple, and they are useful for categorizing structural features related to synthesis conditions and physical properties. However, they only give a qualitative approach and it is difficult to evaluate the physical properties of the objects quantitatively.

The purpose of this paper is to construct three-dimensional structural models composed of primary particles, which are fitted to SAXS experiments on the basis of reverse Monte Carlo (RMC) simulation (McGreevy & Pusztai, 1988 ▸). For the simulation study, we prepared cells of certain sizes (100, 200 and 300 nm) to investigate the effect of cell size when simulating the typical structure of the target materials. These cells contain a huge number of primary particles (more than 100 000 particles in the 300 nm cell) and their positions are moved according to the rules of reverse Monte Carlo simulation. The deviation of the simulation from the experiment is defined by the weight function *w*_*p*_ as
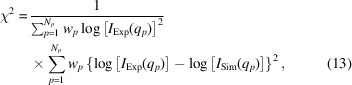
where *I*_Exp_(*q*_*p*_) and *I*_Sim_(*q*_*p*_) are the experimental and simulated scattering intensities, respectively, at wavenumber *q*_*p*_. After movement of a particle position, the newly calculated χ′ is compared with the value before the movement χ. When χ′ < χ, the new configuration is accepted.

**Figure 2:**
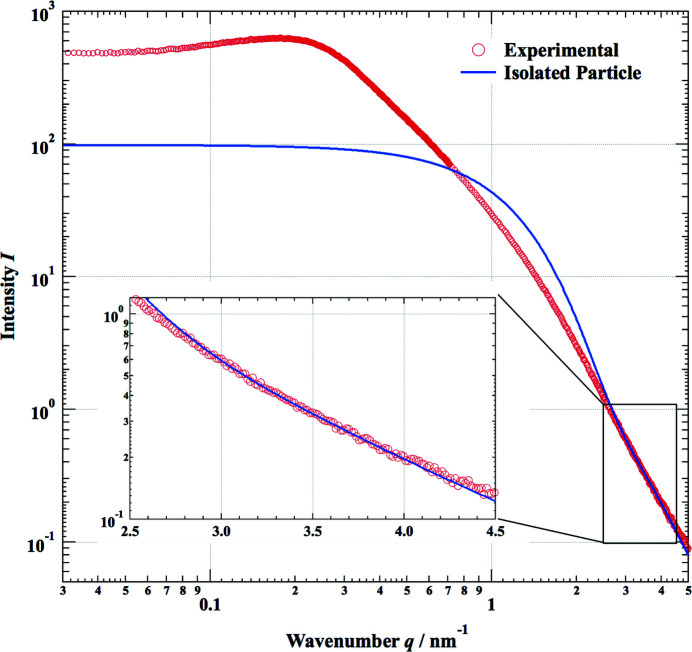
Estimation of the size distribution of the primary particles by comparing the scattering intensity of the isolated-particle simulation and experimental data in the *q* regime from 2.5 to 4.5 nm^−1^.

The SAXS pattern *I*(*q*) for a silica aerogel (SP-30; JFCC, Japan) was collected using a high-performance semiconductor detector (Rigaku HyPix-6000) on a laboratory SAXS measurement system (Rigaku NANOPIX). The X-rays from a high-brilliance point-focus X-ray source (Rigaku MicroMax-007HFMR) were focused and collimated with a multilayer confocal mirror (Rigaku OptiSAXS) and low parasitic scattering pinhole slits (Rigaku ClearPinhole). The specimen was sheet like with a thickness of 1 mm. The SAXS pattern was collected in transmission geometry without any sample treatment. Two data sets measured with different camera lengths of 1400 and 350 mm were combined. The specific features of the SAXS pattern are similar to those from previous studies, but we obtained a wider *q* range, from 0.03 to 5 nm^−1^ with a dynamic range close to 10^4^, as shown in Fig. 2 ▸. As a first step, in order to determine the average diameter 〈*d*〉 of the primary particle, we simulated the SAXS pattern of an isolated particle having some size distribution. This was compared with that of the measured pattern in the higher-*q* regime (2.8 < *q* < 4.3 nm^−1^), as shown in the inset of Fig. 2 ▸. The average diameter was found to be *d* ≃ 2.7 nm, with mean distribution δ*d*/*d* = 0.28. In the lower-*q* regime, the observed SAXS intensity (red circles) is much higher than the calculated SAXS pattern of the isolated particle (solid line). The main objective of the present study is to determine whether we could simulate the observed SAXS intensity on the basis of such a small primary particle size. To calculate equation (12), we set the parameter *R* = *L*/2, which means that the second term in equation (12) is omitted. The resolution Δ*q* of the instrument is 0.0064 nm^−1^, calculated by equation (11) with a spot size at the detector δ_D_ = 0.44 mm and camera length *C*_L_ = 1400 mm. In the RMC simulation procedure, we have adopted a weight parameter in equation (13) of 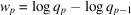, where *q*_*p*_ is the scattering vector value of the *p*th experimental data point. This means equal weight for a log *q* scale rather than a linear *q* scale. Adopting this weight parameter is effective for fitting the low-*q* regime of the SAXS pattern and for constructing large-scale aggregate structures.

**Figure 3:**
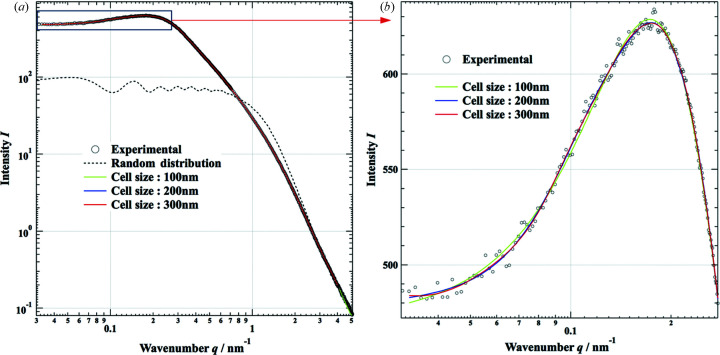
(*a*) Simulated small-angle scattering patterns based on the present SAXS–RMC technique with cell sizes of 100, 200 and 300 nm. The case with randomly positioned primary particles is also shown (dashed line). (*b*) Differences are noted for a cell size of 100 nm (green line) in the low-*q* regime.

The density of the measured aerogel is estimated to be 0.123 g cm^−3^ by comparing the X-ray absorption of the specimen with that of amorphous silica (2.2 g cm^−3^). The volume fraction of the primary particles *v*_f_ is 0.056 and the total numbers of primary particles, *N*, were determined according to the volume fractions to be 4381, 34 983 and 117 846 for simulation cell sizes of 100, 200 and 300 nm, respectively. Fig. 3 ▸(*a*) shows simulated SAXS patterns from the initial arrangement where particles are randomly positioned (dashed line) and after optimization of the particle arrangement by performing RMC fitting with the three cell sizes (green, blue and red lines), together with the experimental data (black circles). In the case of randomly positioned primary particles, the intensity profile is similar to that of an isolated particle as shown in Fig. 2 ▸. This means that the average particle density is very low and the interparticle distance is large enough that we can neglect correlations between particles when they are randomly positioned. The results of RMC fitting with the three cell sizes, 100, 200 and 300 nm, and the observed SAXS pattern overlap with the experimental data, as shown in Fig. 3 ▸. The RMC simulation was repeated until χ^2^ defined by equation (13) decreased to a small value (χ^2^ < 10^−6^) and remained almost unchanged during 100 trials. The total number of trials was more than 7 × 10^7^, with the positions of ten particles moving at once in the case of the 300 nm cell size.

**Figure 4:**
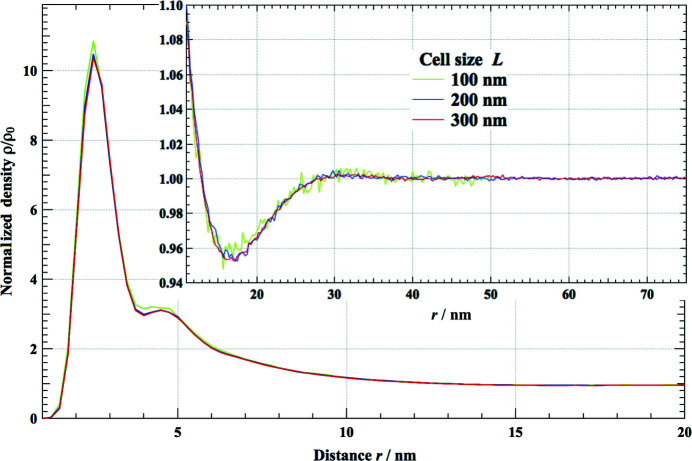
The normalized density distribution between the primary particles, which tends toward 1 in the long-distance regime as shown in the inset. Small differences are observed for the cell sizes of 100 and 200 nm.

The simulated intensity in the low-*q* regime (*q* < 0.5 nm^−1^) has increased about tenfold from that of the initial randomly positioned primary particles. This suggests that large-scale structures are successfully configured by rearranging the positions of the primary particles to fit the experimental SAXS pattern. We also studied what would be an adequate cell size to configure the correct aggregating structure to match the obtained SAXS pattern. Fig. 3 ▸(*b*) shows an enlargement of Fig. 3 ▸(*a*) in the low-*q* (< 0.3 nm^−1^) regime. Small deviations from the experimental SAXS pattern can be recognized with a cell size of 100 nm in this regime. This may be because the aggregated structures of the system could not be configured with a 100 nm cell size. This is also seen in the graph of the density function ρ(*r*) shown in Fig. 4 ▸, where the result for the 100 nm cell is a little different from those obtained with cell sizes of 200 and 300 nm. This suggests a cell size *L* ≥ 200 nm is preferable to configure the appropriate structure of the aerogel to match the observed SAXS pattern down to *q* = 0.03 nm^−1^.

## 4. Comparison with TEM image and pore-size analysis by gas adsorption method   

**Figure 5:**
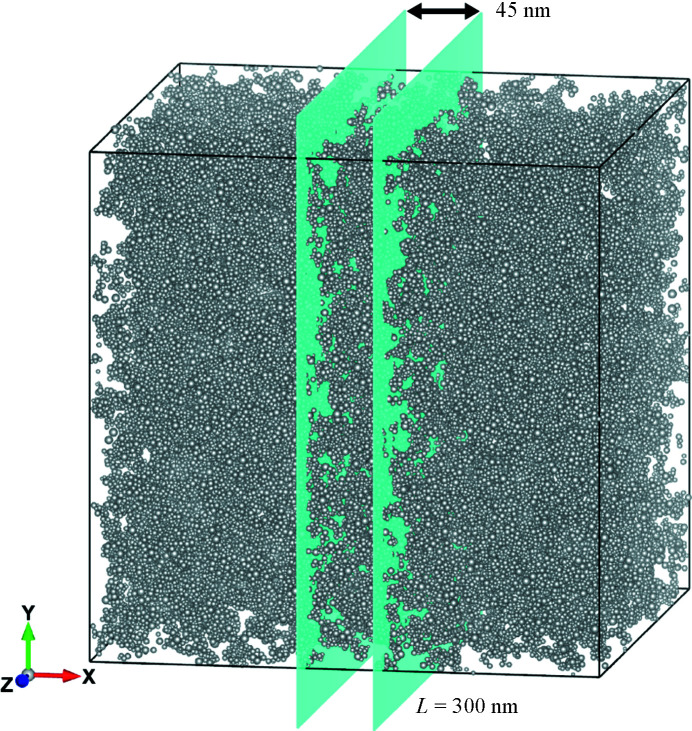
The simulated primary particle configuration for the cell size of 300 nm.

**Figure 6:**
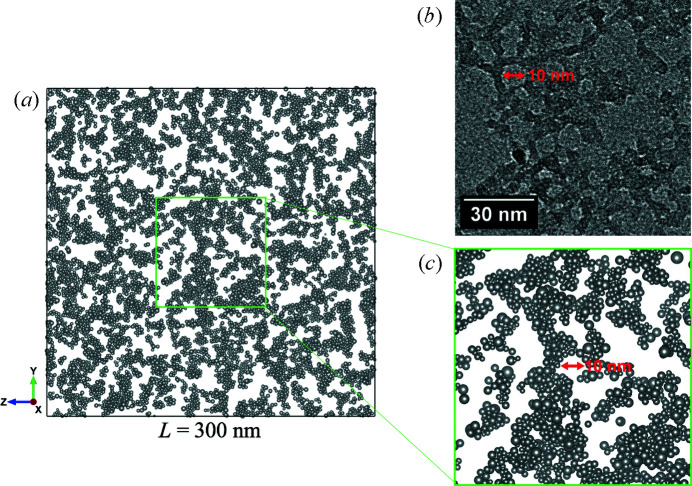
(*a*) A 45 nm thick slice of the primary particle configuration. (*b*) A TEM image of the same sample. (*c*) A magnified image of the primary particle configuration of the same size as the TEM image.

The obtained three-dimensional structure model, which was visualized with the computer program *VESTA* (Momma & Izumi, 2011 ▸), is shown in Fig. 5 ▸, and a slicing image (45 nm thick) is presented and compared with a TEM image in Fig. 6 ▸. Exact comparison between these two images is not easy, but similar characteristic pore sizes of 10 nm can be found in both the TEM image and the slicing image. Note that the real-space simulation matched with the experimental SAXS pattern is able to create a reasonable three-dimensional structure from the primary particles, comparable to the TEM image. Moreover, specimens for TEM should be prepared to be very thin, meaning only a two-dimensional structure can be obtained. On the other hand, SAXS can be measured without any sample preparation and a three-dimensional structure can be simulated using the currently presented RMC simulation.

The three-dimensional configuration of the primary particles obtained by SAXS enables us to investigate the size and connectivity of the pores, key features in the mobility of gas molecules and the thermal conductivity of an aerogel. We analyzed the pore-size distribution through the following procedure.

**Figure 7:**
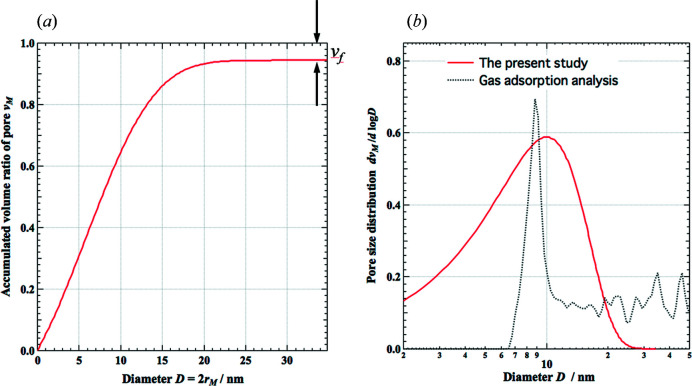
(*a*) The accumulated pore volume of the simulated primary particle configuration. (*b*) The pore-size distribution of the simulated primary particle configuration compared with that obtained by the gas adsorption method.

(i) Every position in the cell is marked with mesh points with a pitch of *u* nanometres. The number of mesh points in the cell is *M* = (*L*/*u*)^3^. We used *u* = 1 nm in the present analysis.

(ii) All the mesh points are classified by a distance *r*_S_ from the surface of the nearest primary particle.

(iii) The number of mesh points *p*(*r*_S_) classified with *r*_S_ is converted to a volume ratio *v*(*r*_S_) = *p*(*r*_S_)/*M*.

(iv) The accumulated volume ratio *v*_*M*_(*r*_S_ ≤ *r*_*M*_) is plotted versus 2*r_M_* in Fig. 7 ▸(*a*), where *D* = 2*r_M_* corresponds to the diameter of the pores. It is obvious that 1 − *v*_*M*_ becomes the volume fraction *v*_f_ of the primary particles asymptotically with increasing *D*.

(v) The pore-size distribution is calculated by the logarithmic derivative of *v*_*M*_ by log*D*, d*v_M_*/d(log*D*), and the result is shown in Fig. 7 ▸(*b*).

To compare with the present simulation, gas adsorption analysis was performed for the same sample, and the result is also shown in Fig. 7 ▸(*b*). The presented gas adsorption measurement was not sensitive to very small pores (*D* < 6 nm) and a discrepancy with the current SAXS–RMC study is noted in this region. However, the mode of the pore size evaluated by the current study (10 nm) agrees well with that obtained by gas adsorption analysis (9 nm). A typical pore size of 10 nm is also found from the TEM observation, as shown in Fig. 6 ▸. This means that the present study can detect the characteristic size of the nanometre-scale structure in the measured aerogel.

## 5. Discussion   

**Figure 8:**
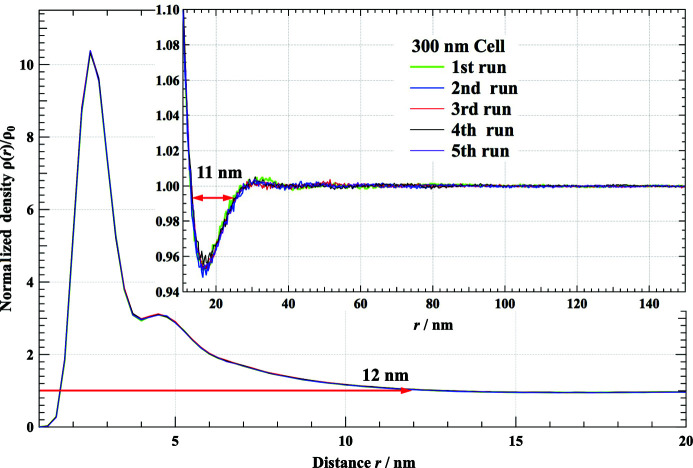
The repeatability of the normalized density function derived from five RMC runs from independent random configurations of primary particles.

We have proposed a SAXS–RMC study to construct a complex aggregated structure from a vast number of simple primary particles. The observed SAXS pattern was well reproduced by the present simulation and the characteristic size of the constructed structure (10 nm) is in fairly good agreement with that observed by TEM and the gas adsorption method. However, the model structure is probably not unique, because the observed SAXS pattern is simple and does not have enough information to determine all the positions of the numerous constituent primary particles. Therefore, we have to interpret the result statistically. One of the key statistical results of the system can be expressed by the density function ρ(*r*). This value has been calculated from SAXS–RMC simulation runs starting from five initial independent random structures, as shown in Fig. 8 ▸. These five density profiles, which started from different initial structures, are almost identical. The obtained density function can be interpreted as follows. The first and second peaks at 2.5 and 4.5 nm, respectively, correspond to the first and second neighbors. The continuing ρ(*r*) > ρ_0_ area up to 12 nm, indicated in Fig. 8 ▸, is interpreted to be aggregated clusters. On the other hand, the small valley with ρ(*r*) < ρ_0_ at a distance from 13 to 24 nm may correspond to pore areas, and the width of the low-density area is about 11 nm, as indicated in Fig. 8 ▸. This is close to the estimated pore size of 10 nm, as mentioned in the previous section. In the next distance range up to 100 nm, very small density oscillations can be recognized. At distances greater than 100 nm, ρ(*r*) tends very close to ρ_0_. This is consistent with our assumption expressed in equation (8). The length scales obtained by the ρ(*r*) function discussed above correspond to the typical structural scale of the observed aerogel.

**Figure 9:**
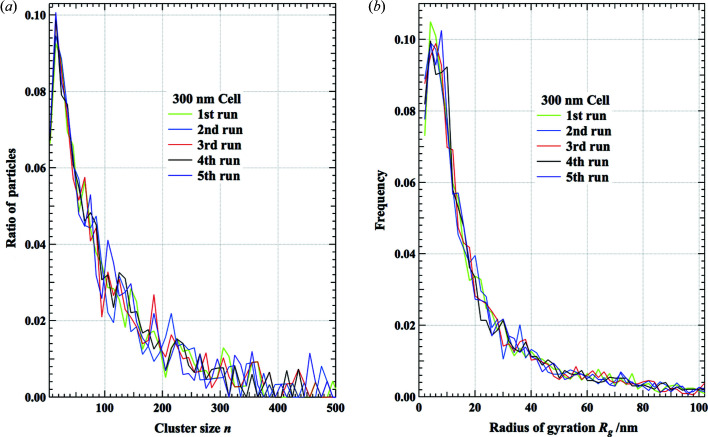
The results of cluster size and shape analysis for five independent RMC runs. (*a*) Cluster size *n* versus ratio of particles that constitute the cluster. (*b*) The distribution of the radius of gyration *R*_g_.

The purpose of the present study is not only to obtain a one-dimensional density function ρ(*r*) but also to extract statistical features of the three-dimensional structure. The pore-size distribution shown in Fig. 7 ▸ is an important characteristic of porous materials (Baychev *et al.*, 2019 ▸) and it cannot be obtained without knowing the real-space structure. The results of the pore-size distributions are almost identical for the five independent RMC runs, as is the calculation of ρ(*r*) (not shown here because the differences between the curves cannot be discerned by the human eye). The other interesting structural feature is the statistics of clusters formed from primary particles. We have extracted contacting particle clusters and classified them according to the number *n* of constituent particles and the radius of gyration *R*_g_. The obtained cluster size and frequency distribution of *R*_g_ with the 300 nm cell size for five independent RMC runs are shown in Fig. 9 ▸. The number *n* peaks at 15 particles and varies widely from a few tens to five hundred, as shown in Fig. 9 ▸(*a*). *R*_g_, which peaks around 4–8 nm, also has a wide distribution from a few nanometres to more than 100 nm, as shown in Fig. 9 ▸(*b*). In both cases, the distributions of the five runs show very similar patterns; even statistical variations in the individual curves are visible. Therefore, the resultant particle configurations of those runs can be recognized as having the same structural features and the present real-space modeling is valid in this sense.

## 6. Conclusion   

We have simulated a three-dimensional aggregated structure from simple primary particles on the basis of SAXS data by the RMC method. The resultant typical structural features are consistent with those observed by TEM and gas adsorption. The constructed three-dimensional structure allows us to calculate the physical properties of the system, for example the transport coefficient of gas diffusion and the thermal conductivity of an aerogel. Similar complex systems such as catalysts and catalytic supports (Kakinuma *et al.*, 2020 ▸) and mesoporous polymers (Samitsu *et al.*, 2013 ▸) have also been extensively studied. We believe the present SAXS–RMC method could allow the evaluation of various critical characteristics of these materials.
